# Discovery of highly reactive self-splicing group II introns within the mitochondrial genomes of human pathogenic fungi

**DOI:** 10.1093/nar/gkab1077

**Published:** 2021-11-25

**Authors:** Tianshuo Liu, Anna M Pyle

**Affiliations:** Department of Molecular, Cellular and Developmental Biology, Yale University, New Haven, CT, 06520, USA; Department of Molecular, Cellular and Developmental Biology, Yale University, New Haven, CT, 06520, USA; Department of Chemistry, Yale University, New Haven, CT, 06520, USA; Howard Hughes Medical Institute, Yale University, New Haven, CT, 06520, USA

## Abstract

Fungal pathogens represent an expanding global health threat for which treatment options are limited. Self-splicing group II introns have emerged as promising drug targets, but their development has been limited by a lack of information on their distribution and architecture in pathogenic fungi. To meet this challenge, we developed a bioinformatic workflow for scanning sequence data to identify unique RNA structural signatures within group II introns. Using this approach, we discovered a set of ubiquitous introns within thermally dimorphic fungi (genera of *Blastomyces*, *Coccidioides* and *Histoplasma*). These introns are the most biochemically reactive group II introns ever reported, and they self-splice rapidly under near-physiological conditions without protein cofactors. Moreover, we demonstrated the small molecule targetability of these introns by showing that they can be inhibited by the FDA-approved drug mitoxantrone *in vitro*. Taken together, our results highlight the utility of structure-based informatic searches for identifying riboregulatory elements in pathogens, revealing a striking diversity of reactive self-splicing introns with great promise as antifungal drug targets.

## INTRODUCTION

Pathogenic fungi place a severe burden on the health care system, and yet we know little about the biology of these ubiquitous organisms. It is estimated that the mortality rate of fungal diseases is comparable to that of tuberculosis and HIV (1). Endemic dimorphic mycoses, especially those within the order of Onygenales, are of particular concern due to their ability to infect otherwise healthy individuals, and they are considered primary human pathogens (2,3). Thermal dimorphism refers to the ability of certain fungi to change their morphology from hyphal to yeast (or spherules in the case of *Coccidioides*) upon temperature change. It is estimated that three thermally dimorphic fungi endemic in North America (*Blastomyces, Coccidioides* and *Histoplasma*) cause >650 000 new infections annually, 50 000 of which are life-threatening (3,4). These three fungi cause blastomycosis, coccidioidomycosis and histoplasmosis, respectively, resulting in fever and pneumonia, along with internal tissue and organ infections in many cases (5). In addition to relatively common fungal infections, lethal classes of rare fungal pathogens are becoming increasingly problematic in both immunocompromised and healthy individuals. For instance, the cerebral chromoblastomycosis caused by the melanin-forming black fungus (dematiaceous fungus) *Cladophialophora bantiana* has a mortality rate of 65% (6).

Despite an increasing fungal disease burden within the patient population, clinical options for managing fungal diseases remain limited. The inherent human toxicity of existing antifungal drugs, along with the gradual acquisition of drug resistance, has further restricted viable treatment options (7). For example, the pathogenic (yeast/spherule) form of thermally dimorphic fungi within the order of Onygenales is inherently resistant to echinocandins, which are considered as the most well-tolerated antifungals (8). Moreover, *Histoplasma capsulatum* is an intracellular pathogen that resides in macrophages, thereby further complicating antifungal development due to issues with efficient drug penetration and host cell toxicity. In light of these therapeutic challenges, it is crucial to develop next-generation antifungals that target novel, fungal-specific pathways.

We recently developed a new class of target-selective small-molecule inhibitors that are potent antifungal agents (‘intronistats’) (9). These molecules target a unique feature of fungal RNA metabolism: a dependence on self-splicing group II introns. Fungal group II introns usually reside in mitochondrial housekeeping genes and their autocatalytic removal from pre-mRNA transcripts is required for proper mitochondrial protein synthesis (10,11). We have demonstrated that targeting the group IIB intron within the *Candida parapsilosis* mitochondrial *cox1* (cytochrome *c* oxidase subunit 1) gene leads to potent and selective growth inhibition of this fungus (9).

However, it has been difficult to extrapolate and generalize this pharmacological approach because we know little about the location, structures and subtypes of group II introns in therapeutically important fungi. The identification of novel group II introns within pathogen genomes has been hampered by the fact that group II introns do not display extensive sequence conservation. For this reason, conventional group II intron search strategies have focused on identification of reverse-transcriptase (RT) proteins known as ‘maturases’ that are encoded within certain group II intron subtypes (12). While this approach succeeds in finding certain RT-encoding group II introns, it fails if a given intron lacks an internal open reading frame (ORF) that encodes conserved endonuclease or reverse transcriptase (RT), or if the intron-encoded RT has degenerated during evolution, which is common for organellar group II introns typical of eukaryotic organisms. Unlike the variability of intronic ORFs, RNA structural signatures of group II introns are highly conserved. For example, semi-conserved 5′ and 3′ structural features have been previously employed to search for group II introns (13). We decided to use another well-conserved structural feature, namely intron domain 5 (D5), a 34-nt stem-loop containing highly conserved nucleotides and a bulge loop that anchors the catalytic core (14,15) to perform the search. Additional secondary and tertiary structural features within group II introns are almost invariant, providing the information needed to confirm and fold group II introns that are mined from primary genomic RNA sequence (16).

Here, we describe an RNA structure-guided approach for discovering novel group II introns in fungal organisms and for defining their functional architecture. This approach has enabled us to discover structurally well-defined, biochemically reactive group II introns in various human pathogenic fungi. Furthermore, we show that these reactive introns are readily inhibited by a small molecule that targets RNA tertiary structure. Our results reveal a diverse and previously invisible intron landscape within human pathogenic fungi, thereby facilitating the future development of group II introns as novel antifungal drug targets for the treatment of severe systemic fungal infections.

## MATERIALS AND METHODS

### Mitochondrial genome retrieval and group II intron analysis

Reference mitochondrial genomes of human pathogenic fungi were retrieved from NCBI GenBank database (the accession numbers of reference genomes used in the analysis are listed in Supplementary Table S1). To survey for the intron presence and location of intron D5, the data were analyzed using RNAweasel pipeline (https://megasun.bch.umontreal.ca/cgi-bin/RNAweasel/RNAweaselInterface.pl) (14). For mitogenomes containing group II introns, the mitochondrial genes and introns were annotated using MFannot pipeline (https://megasun.bch.umontreal.ca/cgi-bin/dev_mfa/mfannotInterface.pl), and the annotations were further subject to manual inspection to validate intron boundaries and flanking gene identity.

### Secondary structure prediction and visualization

The secondary structures were predicted using the mfold webserver (17) with constraints (listed in Supplementary Table S2) and were output in the connection table format. RNAviz software (18,19) was subsequently used to generate secondary structure diagrams.

### DNA constructs and RNA preparation

DNA fragments containing a T7 promoter, 60-nt flanking exons, each intron and a terminal BamHI cleavage site were commercially synthesized (Blue Heron Biotech) and cloned into the pUCminusMCS vector. For *B*.*d*.LSU.I1, *C.i*.LSU.I3 and *C.i*.SSU.I1 introns, the original intron domain 4 sequence (containing intronic ORF sequence) was reduced to a simple stem-loop capped with UUCG tetraloop (intron sequences after ORF deletion are listed in Supplementary Table S2).

The plasmids containing the sequences of the intron self-splicing constructs (denoted as pLTS101 for *H.c*.LSU.I1 intron, pLTS103 for *C.i*.LSU.I3 intron, pLTS104 for *C.i*.SSU.I1 intron and pLTS105 for *B*.*d*.LSU.I1 intron) were linearized using BamHI (New England Biolabs) to generate the DNA template for *in vitro* transcription.

Radiolabeled precursor RNA transcripts were prepared using 5 μg of linearized plasmid and T7 RNA polymerase (expressed and purified in-house) in a transcription buffer containing 40 mM Tris-HCl pH 8.0, 10 mM NaCl, 15 mM MgCl_2_, 2 mM spermidine, 0.01% Triton X-100 and 10 mM DTT. 3.6 mM of each NTP (except UTP) and 1 mM of UTP along with 50 μCi of [α-^32^P]-UTP (PerkinElmer) were added for the transcription. The reaction was incubated at 37°C for 1.5 h, followed by purification on a 5% denaturing polyacrylamide gel. The band corresponding to precursor RNA was cut and eluted overnight in a gel elution buffer (10 mM MOPS-NaOH pH 6.0, 300 mM NaCl and 1 mM EDTA). The RNA transcripts were ethanol precipitated and resuspended in a RNA storage buffer (6 mM MES-NaOH pH 6.0). The RNA stock solution was aliquoted and stored frozen.

### Self-splicing assay and small molecule inhibition assay

For the self-splicing assay, body-labeled precursor RNA (5 nM final concentration) was incubated in 40 mM NH_4_-HEPES pH 7.5 at 90°C for 1 min, followed by incubation at 37°C for 10 min. Monovalent salt stock solution was then added. For reaction with PEG-8000, 50% (w/v) stock solution was also added to the final concentration of 10% (w/v). After incubation at 37°C for 10 min, the splicing reaction was initiated by adding 2 μl of 10× MgCl_2_ stock solution (the final magnesium ion concentrations are indicated in the figures) to a final volume of 20 μl and incubated at 37°C. To monitor reaction time courses, 1 μl was removed from the reaction mixture at specific time points and quenched by mixing with 2× formamide loading buffer (72% (v/v) formamide, 10% sucrose, 0.2% bromophenol blue dye, 0.2% xylene cyanol dye and 50 mM EDTA) and placing on ice. The timepoint samples were then loaded on to a 5% denaturing polyacrylamide gel to resolve individual bands.

For the small molecule inhibition assay, body-labeled intron precursor RNA (5 nM final concentration) was incubated with 2 μl of 10× small molecule stock solution (final DMSO% in the reaction was 10% (v/v)). The reaction buffer containing 40 mM NH_4_-HEPES pH 7.5 and 2 mM MgCl_2_ was used for the assay involving the *C.i*.LSU.I3 and *C.i*.SSU.I1 introns and the buffer containing 40 mM NH_4_-HEPES pH 7.5, 150 mM NH_4_Cl and 10 mM MgCl_2_ was used for the *H.c*.LSU.I1 intron. Total reaction volume was 20 μl and the reaction was incubated 37°C. Time points were taken and analysis was done as aforementioned.

### Data analysis

Gels containing the separated reaction products were dried and exposed to phosphor screens overnight. The screens were scanned using Typhoon FLA9500 phosphorimager (GE Healthcare) or Typhoon RGB Biomolecular imager (Cytiva). Individual bands were quantified using ImageQuant 8.2 software (GE Healthcare). The band intensity was corrected for the uridine content of corresponding species and internally calibrated by calculating the fraction of each species in the reaction.

The precursor depletion time course data were analyzed and plotted using Kaleidagraph 4.5.4 software (Synergy Software). Reactions with monophasic kinetic behavior were fitted into Equation (1).

(1) $$\begin{equation*}{f_{{\rm precursor}}} = {f_{_{{\rm active}}}}\cdot{e^{ - {k_{{\rm obs}}}}}^t + {f_{{\rm inactive}}}\end{equation*}$$

where *f*_precursor_ is the fraction of precursor, *f*_active_ is the fraction of actively converting precursor, *k*_obs_ is the observed rate and *f*_inactive_ is the fraction of non-converting precursor.

Reactions with biphasic kinetics were fitted into Equation (2).

(2) $$\begin{equation*}{f_{{\rm precursor}}} = {f_{{\rm fast}}}\cdot{e^{ - {k_{{\rm fast}}}t}} + {f_{{\rm slow}}}\cdot{e^{ - {k_{{\rm slow}}}t}}\end{equation*}$$

where *f*_precursor_ is the fraction of precursor, *f*_fast_ is the fraction of fast converting precursor, *k*_fast_ is the observed rate of fast converting precursor, *f*_slow_ is the fraction of slowly converting precursor and *k*_slow_ is the observed rate of slowly converting precursor

All reaction time courses were performed in triplicates and error bars in the kinetic plots were reported as standard errors of the mean (S.E.M.).

To determine the *K*_i_ value for small molecule inhibition, the observed rate constant (*k*_obs_) was obtained from the precursor depletion time course, plotted against small molecule concentration and fitted to the noncompetitive inhibition model (Equation 3) as previously described (9). *K*_i_ determination was performed in triplicates to ensure reproducibility and all *k*_obs_ values are reported as mean ± S.E.M.

(3) $$\begin{equation*}{k_{obs}}=\frac{{{k_{\max }}}}{{1 + \frac{{[{\rm{I}}]}}{{{K_i}}}}}\end{equation*}$$

## RESULTS

### Mitochondrial group II intron discovery and secondary structure prediction workflow

**Figure 1.**
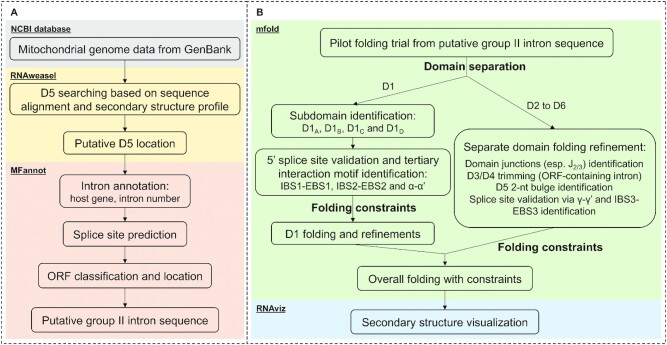
Schematic of the overall workflow. (**A**) The group II intron discovery and annotation flow. Mitochondrial genome data from GenBank are subject to RNAweasel analysis to identify putative intron domain 5 location. The data are subsequently analyzed by MFannot to annotate the intron, predict intron splice sites and identify putative intronic opening reading frame (ORF). The outcome is the putative sequence for a given group II intron. (**B**) Secondary structure prediction workflow. The putative sequence for the group II intron is subject to folding prediction and refinement. In the pilot constraint-free folding trial, intron domains are identified and processed separately. Subsequent separate domain folding refinements yield folding constraints and validate the splice sites. Once folding constraints have been obtained from separate domain folding trials, the intron is folded in whole and the predicted secondary structure is subsequently visualized.

To mine for group II introns within human pathogenic fungi, we first sought to establish a workflow for rapid identification and annotation of new introns (Figure 1A). To that end, we retrieved mitochondrial genomes (mitogenomes) from GenBank and used RNAweasel (14) to search for the conserved 34-nt group II intron D5 based on both its primary sequence conservation and secondary structural features (this search engine is tolerant to small variations of D5, as described in (14)). Once a candidate D5 region was identified, the corresponding genome was further analyzed by MFannot to predict intron/exon boundaries and to annotate the intron. Intron/exon boundaries were predicted based on intron splice site selection rules and exon sequence alignment with reference mitochondrial genomes in the database (14). The actual genes interrupted by the intron were further annotated based on sequence homology and the intron number was used to indicate the order of the intron within its host gene. MFannot was also used to identify putative intronic open reading frames (ORFs). This analysis yielded identification, annotation and putative sequence of the group II introns.

We next aimed to set up a secondary structure prediction workflow (Figure 1B) to evaluate the bioinformatic predictions of intron/exon boundaries and to predict the secondary structure of newly identified group II introns. To this end, putative group II intron sequences were subject to a pilot, constraint-free folding trial using the mfold webserver (17). Since individual group II intron domains tend to fold autonomously (20), the pilot folding stage was designed to efficiently identify and separate the largest D1 from the rest of the intron (D2 to D6). D1 was previously shown to fold independently, adopting its native structure during the first step of intron folding, subsequently serving as the scaffold for assembly of downstream intron domains (16). The top 5 lowest free energy predictions were manually examined for the existence of D1 basal stem structural features (D1(i) stem, D1(i) loop and D1(ii) stem) and the single-stranded junction region between D1 and D2 (J1/2) to define the boundary of D1. We then focused on the D1 sequence to refine its secondary structure prediction. In the beginning, we sought to establish the five-way junction of D1. The four subdomains of D1 (D1A, D1B, D1C and D1D) were identified and folding constraints were obtained to ensure formation of each subdomain (particularly the short stems of D1A and D1B, which were forced to be base-paired). Along with the D1 basal stem identified from the pilot stage, the five-way junction skeleton of D1 is therefore established. Meanwhile, we searched for three important tertiary interaction motifs, namely IBS1-EBS1 (IBS, intron-binding site; EBS, exon-binding site), IBS2-EBS2 and α-α’ (21,22), as these can be predicted faithfully by covariation in base pairing. Identification of these critical tertiary interaction motifs not only provided folding constraints for the proper formation of the largest D1D subdomain, but also validated the 5′ splice site prediction (as the intron 5′ end is immediately after the IBS1 sequence). We then used the newly defined folding constraints to refine our overall folding model for D1, during which additional known tertiary interaction motifs (such as θ, ϵ and κ (21,22)) were identified and set as constraints when possible. Prior to the folding of D2 to D6, any intronic ORF sequences were removed from their host domain (D3 or D4) to simplify subsequent RNA structure prediction and facilitate the design of self-splicing constructs. The 2-nt bulge in D5 was also forced to be AY as it tends to be incorrectly predicted using thermodynamic parameters alone (23). The domain junction sequences were subsequently identified and set as folding constraints (forced to be single-stranded). Next, we validated the 3′ splice site prediction by examining the presence of γ-γ’ and IBS3-EBS3 base pairs (γ’ is the ultimate nucleotide of the intron; IBS3 is immediately after the γ’ nucleotide and is the first nucleotide of the 3′ exon) (22), which defines the intron/3′exon boundary. Finally, we combined all of the folding constraints to assemble and visualize the entire secondary structure using RNAviz (19).

### Group II introns uncovered in human pathogenic fungi

Having established the intron mining workflow, we then employed it to probe the presence of novel group II introns within the reference mitochondrial genomes of important human pathogenic fungi (Supplementary Table S1). In addition to the intron within the yeast-like opportunistic pathogen *Candida parapsilosis* which was previously identified (9,15), we uncovered new group II introns in pathogens spanning both thermally dimorphic fungi (genera of *Blastomyces, Coccidioides* and *Histoplasma*) and dematiaceous fungi (genus of *Cladophialophora*) (Table 1). The introns insert into both protein-encoding genes (pre-mRNA introns) and mitochondrial ribosomal (mitoribosomal) genes (pre-rRNA introns). The pre-mRNA introns we identified represented a maturase lineage of introns, as all but one intron contain an ORF encoding a maturase-type reverse transcriptase (Supplementary Table S1). However, due to their limited prevalence across different species, these pre-mRNA introns were not further pursued. By contrast, the pre-rRNA introns we identified were widely present and of therapeutic interest. The removal of pre-rRNA introns is a prerequisite for the maturation of functional ribosomes since intron retention prevents association of the two ribosomal subunits (24) and therefore inhibits mitochondrial protein synthesis. It has been shown that a splicing-defective mitoribosomal group II intron leads to senescence and hypovirulence phenotype in the plant fungal pathogen *C. parasitica* (25). Therefore, we chose to perform further analysis on these ubiquitous pre-rRNA introns and examine various aspects of their properties (Table 2). The GC content of these introns is generally ∼30%, similar to the yeast ai5γ intron (27%, D4-excluded sequence) but significantly lower than the well-studied brown algae *P.li*.LSU.I2 intron (43%, D4-excluded sequence) (26,27). Moreover, our analysis further revealed that the intronic ORFs in these examples were highly variable. The mtLSU (mitoribosomal large subunit) introns in *B. dermatitidis*, *C. posadasii*, *C. bantiana*, *C. carrioni* and *H. capsulatum* are ORF-less while the rest of the introns contain intronic ORF encoding LAGLIDADG-type homing endonucleases (LHE). The intronic ORF location was also found to be variable, as it was located in D4 for the first mtSSU (mitoribosomal small subunit) intron in *C. immitis* (*C.i*.SSU.I1) and in D3 for the second mtSSU intron (*C.i*.SSU.I2).

**Table 1.** Prevalence of group II introns within human pathogenic fungi.

				Group II intron insertion site(s)	
Disease	Organism	Classification	Reference genome GenBank accession	pre-mRNA(s)	pre-rRNA(s)
Candidiasis	*C. parapsilosis*	Yeast	NC_005253	*cox1*	/
Blastomycosis	*B. dermatitis*	Dimorphic	GG753566*	*/*	LSU, SSU
Coccidioidomycosis	*C. immitis*		NW_004504306^†^	*cox1*	LSU, SSU
	*C. posadasii*		DS544748^‡^	*cox1*	LSU, SSU
Histoplasmosis	*H. capsulatum*		GG692467^§^	*/*	LSU
Chromoblastomycosis	*C. bantiana*	Dematiaceous	NC_030600	*/*	LSU
	*C. carrioni*		LGRB01000023	*/*	LSU

*strain ATCC 18188, see Supplementary Table S2 for the presence survey in different strains (same below).

^†^strain RS.

^‡^strain CPA0001.

^§^strain H143.

**Table 2.** Properties of the newly discovered mitochondrial pre-rRNA introns within human pathogenic fungi.

Order	Organism	Reference genome GenBank accession	Intron	Position	ORF type	ORF location (intronic domain)	GC%
Onygenales	*B. dermatitidis*	GG753566	*LSU*.I2	11143…11940	ORF-less		28
			*SSU*.I1	49637…1080	LHE	3	31*
	*C. immitis*	NW_004504306	*LSU*.I3	34743…35943	LHE	4	32*
			*SSU*.I1	21647…24035	LHE	4	31*
			*SSU*.I2	24193…25096	LHE	3	33*
	*C. posadasii*	DS544748	*LSU*.I3	31767…32543	ORF-less		31
			*SSU*.I1	18675…20362	LHE	3	32*
	*H. capsulatum*	GG692467	*LSU*.I1	27672…27045^†^	ORF-less^‡^		29
Chaetothyriales	*C. bantiana*	NC_030600	*LSU*.I2	3634…4375	ORF-less		26
	*C. carrioni*	LGRB01000023	*LSU*.I1	19501…20192	ORF-less		29

*GC content is calculated based on ORF-excluded sequence.

^†^Both the host gene and the intron are located at the reverse compliment Crick strand.

^‡^Strain variation is observed, see Supplementary Figure S2 for detail.

Abbreviations: LHE, LAGLIDADG-type homing endonuclease; ORF, open reading frame.

After identification of novel pre-rRNA introns in the reference strains, we then sought to determine if they are widely present in different strains of the same species. This question is of critical importance because the prevalence of a given intron among different strains is a prerequisite for prioritizing these introns as targets for drug screening. We applied a BLASTN search to evaluate the intron prevalence in all strains for which whole genome assembly data were available, finding that the vast majority of strains contain corresponding introns (Supplementary Table S3). Moreover, we noticed a striking strain variance in *H. capsulatum*. In the African strain (H88 and H143; *H. capsulatum var. duboisii*), the intron is ORF-less while in the North American strain (NAm1; *H. capsulatum var. capsulatum*), the intron contains the ORF encoding for LHE (Supplementary Figure S1). This observation again reveals the variable nature of intronic ORFs due to a relative lack of selection pressure and highlights the advantage of using conserved RNA structural motifs for mining group II introns over approaches that search for intron-encoded proteins (IEPs) (12).

**Figure 2.**
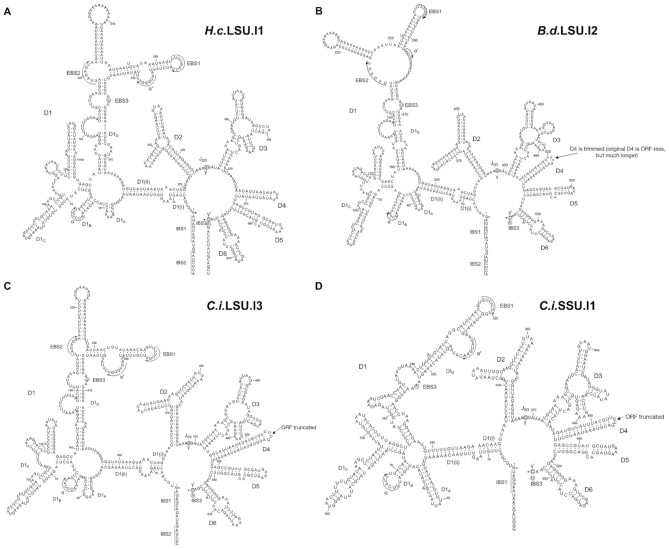
Secondary structure prediction of representative mitochondrial pre-rRNA group II introns newly identified in dimorphic fungi. Important tertiary interactions used as folding constraints (EBS1-IBS1, EBS2-IBS2, α-α’ and γ-γ’) are indicated in the secondary structure diagrams. (**A–C**) The mitochondrial large subunit rRNA introns in *Histoplasma capsulatum* (*H.c*.LSU.I1)*, Blastomyces dermatitidis* (*B.d*.LSU.I2) and *Coccidioides immitis* (*C.i*.LSU.I3), respectively. (**D**) The mitochondrial small subunit rRNA intron found in *Coccidioides immitis* (*C.i*.SSU.I1). The intronic domain 4 of *B.d*.LSU.I2, *C.i*.LSU.I3 and *C.i*.SSU.I1 introns, are reduced to a simple stem-loop capped with UUCG tetraloop.

In the next step, we predicted intron secondary structures (Figure 2 and Supplementary Figure S2), using the workflow described above, to gain further structural insights into the introns we had discovered. All these introns fall into the group IIB1 subclass upon inspection of the predicted secondary structure (28). We found that all the LSU introns insert into the same position (between A2059 and A2060 in the *Escherichia coli* 23S rRNA numbering). By contrast, the SSU introns have two insertion sites (between U788 and U789, U952 and G935 in the *E. coli* 16S rRNA numbering) and represent a unique lineage lacking the IBS2–EBS2 interaction, as previously noticed in other fungi (15,29). With predicted secondary structures in hand, we then prioritized introns with the most well-defined secondary structure profiles for experimental biochemical characterization. The following selection criteria were used to define a well-determined secondary structure: (i) a minimal fraction of loop regions and (ii) a minimum of unconstrained structural elements that are not rigidified by known tertiary interactions.

Four introns, namely *H.c*.LSU.I1, *B.d*.LSU.I2, *C.i*.LSU.I3 and *C.i*.SSU.I1 (Figure 2), met both criteria. These introns are representatives of the newly discovered class of pre-rRNA introns as they come from three important dimorphic fungal genera (*Histoplasma*, *Blastomyces* and *Coccidioides*) and the two introns from *C. immitis* represent the LSU and SSU intron lineage respectively. Hence, we selected them for downstream *in vitro* biochemical characterization.

### Robust self-splicing activity of newly identified fungal group II introns

We then sought to evaluate the self-splicing behavior of the representative introns *in vitro*. Self-splicing precursor molecules were designed and synthesized from DNA templates using T7 RNA polymerase. After purification and isolation by electrophoresis, these precursor molecules were subject to the pilot self-splicing assay to evaluate the magnesium ion dependence of self-splicing (Supplementary Table S4). We noted that they required surprisingly low magnesium ion concentration to become active in self-splicing, indicating superior stability and reactivity. Based on the biochemical profiles obtained from the pilot self-splicing assay, we further narrowed the scope to three introns (*H.c*.LSU.I1, *C.i*.LSU.I3 and *C.i*.SSU.I1) for further investigation. The *B.d*.LSU.I2 intron was not subject to additional biochemical characterization as it has high degree of sequence similarity and demonstrates nearly identical biochemical behavior to the *H.c*.LSU.I1 intron, which is line with the close phylogenetic distance between *H. capsulatum* and *B. dermatitidis* (30).

**Figure 3.**
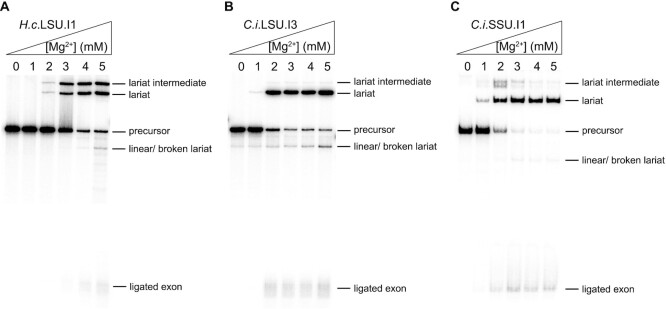
Representative pre-rRNA group II introns readily self-splice under near-physiological conditions. The reaction is performed in the buffer containing 40 mM NH_4_-HEPES pH 7.5, 150 mM NH_4_Cl and 10% PEG-8000 under various magnesium ion concentrations as indicated on top of the gel lanes. After incubation at 37°C for 1 h, the reaction is quenched and loaded onto the 5% polyacrylamide/ 8 M urea gel (as described in Materials and Methods). The splicing gels are shown for (**A**) *H.c*.LSU.I1 intron, (**B**) *C.i*.LSU.I1 intron and (**C**) *C.i*.SSU.I1 intron. The middle part of the gel is not shown as no bands are visible within that region.

To examine overall splicing activity of the new introns, we monitored intron self-splicing under near-physiological conditions of low salt concentration (40 mM HEPES pH 7.5 and 150 mM NH_4_Cl) and 10% PEG-8000 (polyethylene glycerol with an average molecular weight of 8000; to mimic the crowded cellular environment, *vide infra*) at 37°C. The magnesium ion concentration was titrated from 0 to 5 mM (Figure 3). We observed that the *H.c*.LSU.I1 intron readily branches at a magnesium concentration as low as 3 mM, although a significant amount of lariat intermediate accumulates under this condition (Figure 3A), suggesting a facile first step and a slower second step of splicing. The LSU and SSU introns from *C. immitis* appeared more robust, as both of them readily self-splice when the magnesium ion concentration is as low as 2 mM, with minimal accumulation of lariat intermediate (Figure 3B and C). It is notable that little hydrolytic product was observed under these reaction conditions for all three introns assayed, suggesting that they have evolved to favor the branching pathway.

Furthermore, we also evaluated the influence of monovalent cation identity on the self-splicing reaction under the physiologically relevant concentration of 150 mM (Supplementary Figure S3). We observe that potassium stimulates self-splicing almost as efficiently as ammonium ion. Intriguingly, sodium can support the self-splicing reaction but it generally requires slightly higher concentrations of Mg^2+^ for optimal efficiency. This observation is surprising because other group II introns either react inefficiently or become trapped at the intermediate state when sodium is the only available monovalent cation (27,31). It is also notable that with increasing concentration of magnesium ion, the apparent linear intron band intensity went up, which was subsequently determined as a product from lariat breakage instead of from the competing hydrolytic pathway (Supplementary Figure S4).

Taken together, the introns we have identified in pathogenic fungi readily self-splice under near-physiological conditions and react almost exclusively through the branching pathway. These findings indicate that these introns are unusually reactive, as they can fold into catalytically active conformations and accurately position the branch-site adenosine under salt and temperature conditions that are typically insufficient for promoting group II intron catalysis.

### Rapid self-splicing kinetics under near-physiological, mild conditions

Given that the introns are stable and self-splice in a robust fashion, we set out to characterize self-splicing kinetics in order to evaluate overall catalytic efficiency (Supplementary Table S5). By monitoring the depletion of self-splicing precursor molecules as a function of time, we observe that these novel introns self-splice extremely rapidly, at a speed that is even faster than that of model group IIB1 introns splicing facilitated by protein cofactors. For example, the *C.i*.LSU.I3 intron self-splices with an observed rate constant of 0.75 ± 0.029 min^–1^ (150 mM NH_4_Cl and 5 mM MgCl_2_, pH 7.5, 37°C). As a comparison, the observed rate constant of DEAD-box helicase Mss116-promoted ai5γ (LE) intron splicing is 0.052 ± 0.0059 min^–1^ (100 mM KCl and 9 mM unbound MgCl_2_, pH 7.5, 30°C) (32), which is slower by more than an order of magnitude.

**Figure 4.**
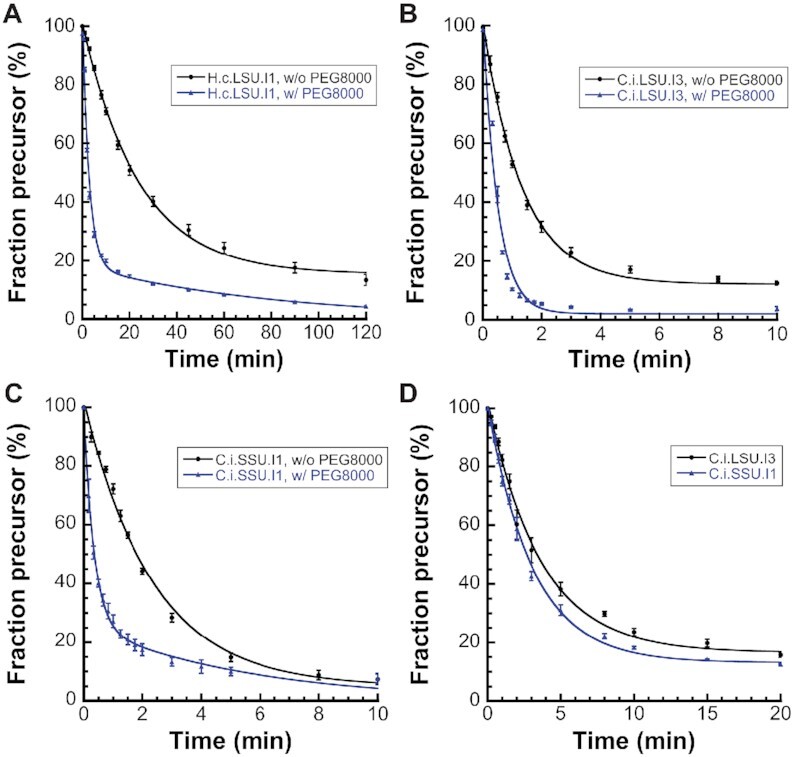
Kinetic characterization of representative novel fungal group II introns. (**A–C**) Precusor depletion time courses without (black circle, •) and with (blue triangle, ▴) 10% PEG-8000. Crowding agent PEG-8000 greatly enhances reaction rate and helps resolving non-converting species under the low-salt condition (40 mM NH_4_-HEPES pH 7.5, 150 mM NH_4_Cl) at 37°C. (A) Time course of precursor depletion for *H.c*.LSU.I1 intron under 10 mM MgCl_2_. The observed rates without and with PEG-8000 are 0.039 ± 0.001 min^–1^ and 0.36 ± 0.04 min^–1^, respectively. (B) Time course of precursor depletion for *C.i*.LSU.I3 intron under 5 mM MgCl_2_ without and with 10% PEG-8000. The observed rates without and with PEG-8000 are 0.75 ± 0.03 min^–1^ and 2.00 ± 0.22 min^–1^, respectively. (C) Time course of precursor depletion for *C.i*.SSU.I1 intron under 5 mM MgCl_2_ without and with 10% PEG-8000. The observed rates without and with PEG-8000 are 0.43 ± 0.02 min^–1^ and 2.94 ± 0.18 min^–1^, respectively. (**D**) Two introns from *C. immitis*, *C.i*.LSU.I3 (black circle, •) and *C.i*.SSU.I1 (blue triangle, ▴) rapidly self-splice under extra mild condition (40 mM NH_4_-HEPES pH 7.5, 0.5 mM MgCl_2_) at 37°C with 10% PEG-8000. The observed rates for *C.i*.LSU.I3 and *C.i*.SSU.I1 intron are 0.28 ± 0.02 min^–1^ and 0.33 ± 0.02 min^–1^, respectively. Data represent the average of *n* = 3 independent experiments; error bars represent standard errors of the mean (S.E.M.).

We then examined the effect of crowding agent PEG-8000 on the self-splicing kinetics. Unlike a cellular environment containing osmolytes and macromolecules that affect RNA folding (33), many *in vitro* biochemical reactions are typically performed in a highly diluted solution environment devoid of those factors. To recapitulate the *in vivo* reaction conditions, crowding agents such as PEG (polyethylene glycerol) have been employed (34,35). One particular member of the PEG family, namely PEG-8000, was previously shown to increase the rate of the *trans* oligo cleavage reaction catalyzed by ai5γ intron-derived ribozyme (36), so we set out to determine if PEG-8000 can accelerate *cis* self-splicing reaction as well. To this end, we monitored the time course of self-splicing with and without PEG-8000 under the lowest magnesium ion concentration that supports rapid self-splicing under both conditions (10 mM for *H.c*.LSU.I1; 5 mM for *C.i*.LSU.I3 and *C.i*.SSU.I1). The precursor depletion time courses (Figure 4A–C) reveal that PEG-8000 significantly accelerates the self-splicing rate constant for all three introns tested and resolves non-converting species observed during reactions of the *H.c*.LSU.I1 and *C.i*.LSU.I3 introns. This stimulatory effect is the most pronounced for the *H.c*.LSU.I1 intron, where the observed precursor depletion rate constant increases nearly 10-fold upon addition of PEG-8000, from 0.039 ± 0.001 min^–1^ to 0.36 ± 0.04 min^–1^ (fast species rate). PEGs with shorter length, such as PEG-2000 and PEG-4000, can also promote the efficiency of self-splicing (Supplementary Figure S5A) but to a lesser extent than PEG-8000. We also monitored the self-splicing reaction of natively purified *H.c*.LSU.I1 intron precursor RNA with and without PEG-8000 (Supplementary Figure S5B). Little difference is noted between natively purified and refolded precursor RNA, confirming that the measured rate constants are insensitive to the type of purification protocol. Moreover, the observed rate constant for the *C.i*.LSU.I3 and *C.i*.SSU.I1 introns achieves 2.00 ± 0.22 min^–1^ and 2.94 ± 0.18 min^–1^ (fast species rate), respectively, in the presence of PEG-8000. To the best of our knowledge, these are the fastest rate constants ever observed for a group II intron self-splicing reaction, and it underscores the importance of stable folding and molecular crowding in promoting catalytic efficiency of group II introns.

Moreover, in the presence of a crowding agent, we found that, under extremely low-salt conditions (monovalent cation in the solution comes solely from the 40 mM NH_4_-HEPES pH 7.5 buffer with no additional ammonium salt), self-splicing of *C.i*.LSU.I3 and *C.i*.SSU.I1 introns occurs even under conditions of sub-millimolar magnesium ion (Supplementary Table S4, entry 11 and 16). To further evaluate the catalytic efficiency under such unusually mild conditions, we monitored the self-splicing time course (Figure 4D) and found that the two introns retain rapid self-splicing efficiency under sub-millimolar (0.5 mM) magnesium, with observed rate constants of 0.28 ± 0.02 min^–1^ and 0.33 ± 0.02 min^–1^ for the *C.i*.LSU.I3 and *C.i*.SSU.I1 introns, respectively. Such rapid rate constants under minimal monovalent and magnesium ion concentrations highlight the unprecedented robustness and catalytic efficiency of the two novel group II introns from *C. immitis*.

Taken together, these results show that molecular crowding can significantly boost efficiency of group II intron self-splicing, which is consistent with previous studies showing the strong stabilizing effect of crowding agents on RNA tertiary structures. Moreover, the two pre-rRNA introns in the pathogenic fungus *C. immitis* stand out for their facile branching and splicing at very low concentrations of monovalent salt and magnesium, and for their unprecedently rapid self-splicing kinetics under these same conditions in the absence of protein cofactors. These observations underscore the importance of investigating catalytic RNA molecules from diverse eukaryotic organisms and they reveal the catalytic potential of group II introns as enzymes.

### Mitoxantrone strongly inhibits intron self-splicing *in vitro*

**Figure 5.**
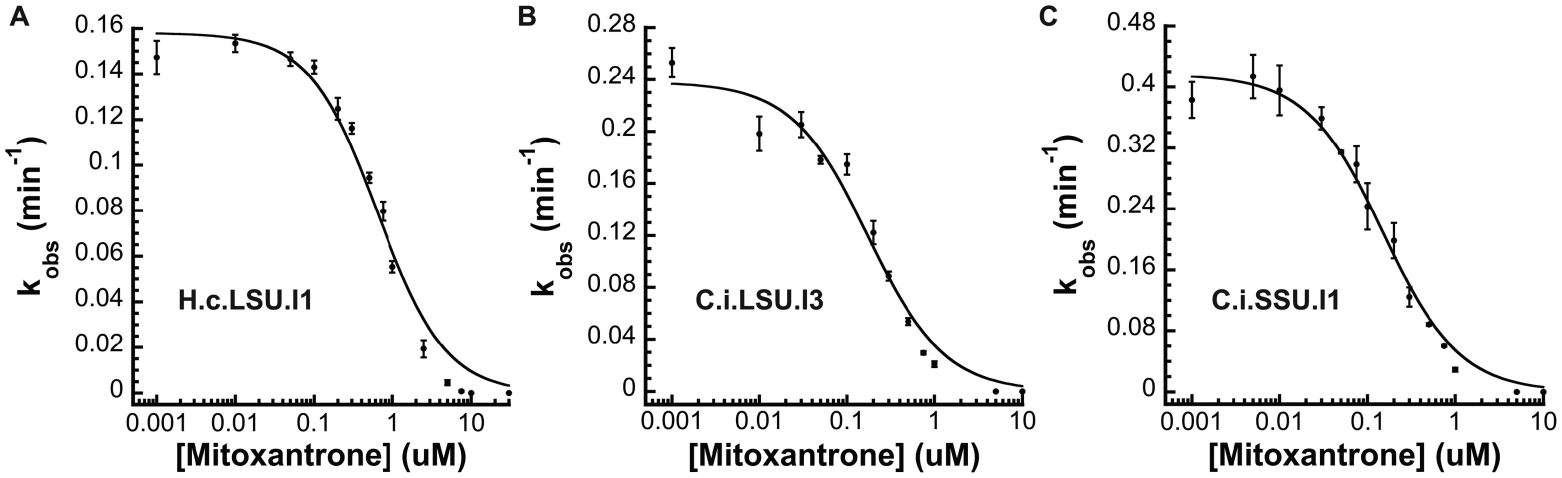
Mitoxantrone potently inhibits the intron self-splicing reaction for all three representative fungal group II introns *in vitro*. The observed rate constants (*k*_obs_) are plotted against compound concentration to give the inhibition constant *K*_i_. The *K*_i_ values are determined to be 0.64 ± 0.08 μM, 0.18 ± 0.03 μM and 0.15 ± 0.01 μM for (**A**) *H.c*.LSU.I1, (**B**) *C.i*.LSU.I3 and (**C**) *C.i*.SSU.I1 intron respectively. Data represent the average of *n* = 3 independent experiments; error bars represent standard errors of the mean (S.E.M.).

To evaluate the targetability of the introns we have identified in pathogenic fungi, we tested whether their self-splicing was sensitive to mitoxantrone, an FDA-approved drug with broad RNA-targeting capability (37–39) known to disrupt stable RNA folds. Indeed, mitoxantrone non-competitively inhibits the intron self-splicing reaction in a dose-dependent manner. The inhibition constants (*K*_i_) were determined to be 0.64 ± 0.08 μM, 0.18 ± 0.03 μM and 0.15 ± 0.01 μM for the *H.c*.LSU.I1, *C.i*.LSU.I3 and *C.i*.SSU.I1 introns, respectively (Figure 5). These sub-micromolar affinity values represent potency levels rivaling that of other well-established small molecule RNA inhibitors (9,40,41). This work demonstrates that the introns are targetable by small molecules, such as mitoxantrone, *in vitro*. Since broadly active ribozyme inhibitors are unusual, our results offer new insights into ribozyme inhibitor design and suggest utility in repurposing existing drugs.

## DISCUSSION

In this study, we have shown that the unique RNA sequence and structural features of group II introns can be used to mine sequence databases for the discovery of new autocatalytic introns, particularly those involved in key biochemical processes within pathogenic organisms. In this case we used the most conserved secondary structural feature of group II intron, D5, to uncover novel group II introns in the mitogenome of human pathogenic fungi, thereby eliminating the need for group II intron discovery protocols based on intron-encoded proteins, which are highly variable. By combining this approach with the known group II intron secondary and tertiary structural determinants, we were able to accurately predict the secondary structure of newly identified introns, which in turn guided our selection of intron targets for experimental characterization. In our case, this structure-based approach eventually led to the discovery of unprecedentedly reactive group II introns in several therapeutically challenging mycoses.

The same approach can be generalized to discover and characterize other highly structured RNA molecules that represent potential drug targets, such as self-splicing group I introns and RNase P RNA, both of which are already identifiable through the RNAweasel pipeline (14). Additionally, it is possible to extrapolate our motif-based discovery approach to various non-fungal genomes as an expedited method for discovering riboregulatory elements. For instance, the -1 PRF (programmed ribosomal -1 frameshift) motif in beta-coronaviruses is highly conserved on the secondary structure level (42), making it possible to pinpoint and predict the secondary structure of -1 PRF from only the sequencing data, which is conceptually akin to the group II intron discovery and secondary structure prediction workflow described here.

While the introns we have discovered are particularly significant because of their potential utility as antifungal drug targets, they are also inherently intriguing because of their unique biochemical and enzymological behavior. They are characterized by unusually robust and efficient self-splicing, which may reflect fungal adaption to a pathogenic lifestyle. For example, magnesium ion is crucial for intron catalytic activity, but it is significantly restricted for the pathogen during infections within the host (43). Perhaps the self-splicing introns in pathogenic fungi have evolved to utilize magnesium efficiently in order to cope with magnesium deprivation in the host. In addition, the ability of those introns to self-splice robustly and rapidly facilitates efficient gene expression even when cognate protein cofactors or magnesium transporters known to promote intron splicing (44,45) are unavailable in a new host environment.

In addition to a possible role in host adaption, it is interesting to postulate the potential involvement of group II intron splicing in the mitochondrial regulatory network of thermally dimorphic fungi that are endemic in North America (*Blastomyces, Coccidioides* and *Histoplasma*), as it might serve as a selection pressure against intron degeneration or loss. It is known that group II introns can act as temperature sensors for post-transcriptional response to temperature cues (46,47). These precedents suggest that group II introns in dimorphic pathogens could also function as temperature sensors, particularly during the unique thermally-induced morphology change of dimorphic fungi.

From a therapeutic perspective, targeting fungal mitochondrial function has become a promising new paradigm in the development of antifungal drugs. A clinical stage mitochondrial functional inhibitor, T-2307, displays potent inhibitory activity against a wide array of pathogenic fungi both *in vitro* and in murine infection models (48,49). In parallel, our lab has demonstrated that mitochondrial group II intron splicing inhibitors can block mitochondrial respiration without concomitant cytotoxicity in mammalian cells, therefore providing a more potent, fungal-specific strategy than conventional electron transport chain inhibitors (50). Here we demonstrate that the unique set of introns we have identified in dimorphic fungi can be readily inhibited by the FDA-approved small molecule drug mitoxantrone. These findings open the possibility of a novel chemical biology strategy to study the functional implications of mitochondrial respiration in this unique class of dimorphic fungi by selectively manipulating their mitochondrial intron splicing. These findings will facilitate the development of next-generation therapeutics against the growing threat of dimorphic fungal pathogens.

Beyond autocatalytic introns, the targeting of other selfish genetic elements also emerges as a new concept in the continuous fight against microbial human pathogens. For example, the fungal pathogen *Cryptococcus* contains an intein within its central spliceosomal protein Prp8 and small molecules inhibiting specific intein splicing might lead to potent anti-*Cryptococcus* activity (51,52). Taken together, the great promise of selfish genetic elements as antimicrobial drug targets warrants ongoing mechanistic functional studies and drug screening efforts to fill the current gap in antimicrobial discovery.

## DATA AVAILABILITY

All data are available in the main text or the supplementary materials. Additional data related to this paper may be requested from the corresponding author.

## Supplementary Material

gkab1077_Supplemental_Files
