# Supplementary material for: Discovery of highly reactive self-splicing group II introns within the mitochondrial genomes of human pathogenic fungi
Source: Nucleic Acids Res. 2021 Nov 25;49(21):12422–32. doi: 10.1093/nar/gkab1077 (PMC8643640; doi:10.1093/nar/gkab1077)
Supplement: gkab1077_Supplemental_Files [file gkab1077_supplemental_files.zip › SI_Liu_NAR_revised_final.pdf]

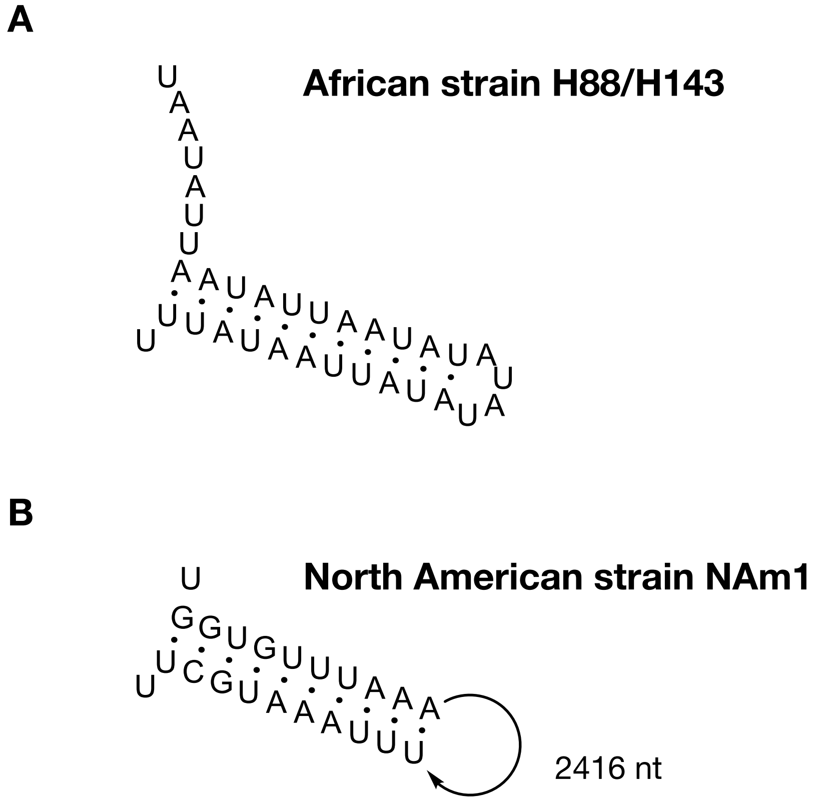

Supplementary Figure 1. Intron D4 variation in different strains of *Histoplasma capsulatum*.
(A) In the African strains H88 and H143 (*H. capsulatum var. duboisii*), the intron D4 is a simple stem-loop consisting of purely A-U base pairs.
(B) In the North American strain NAm1 (*H. capsulatum var. capsulatum*), the intron D4 contains ORF encoding for the LAGLIDADG-type homing endonuclease (LHE).

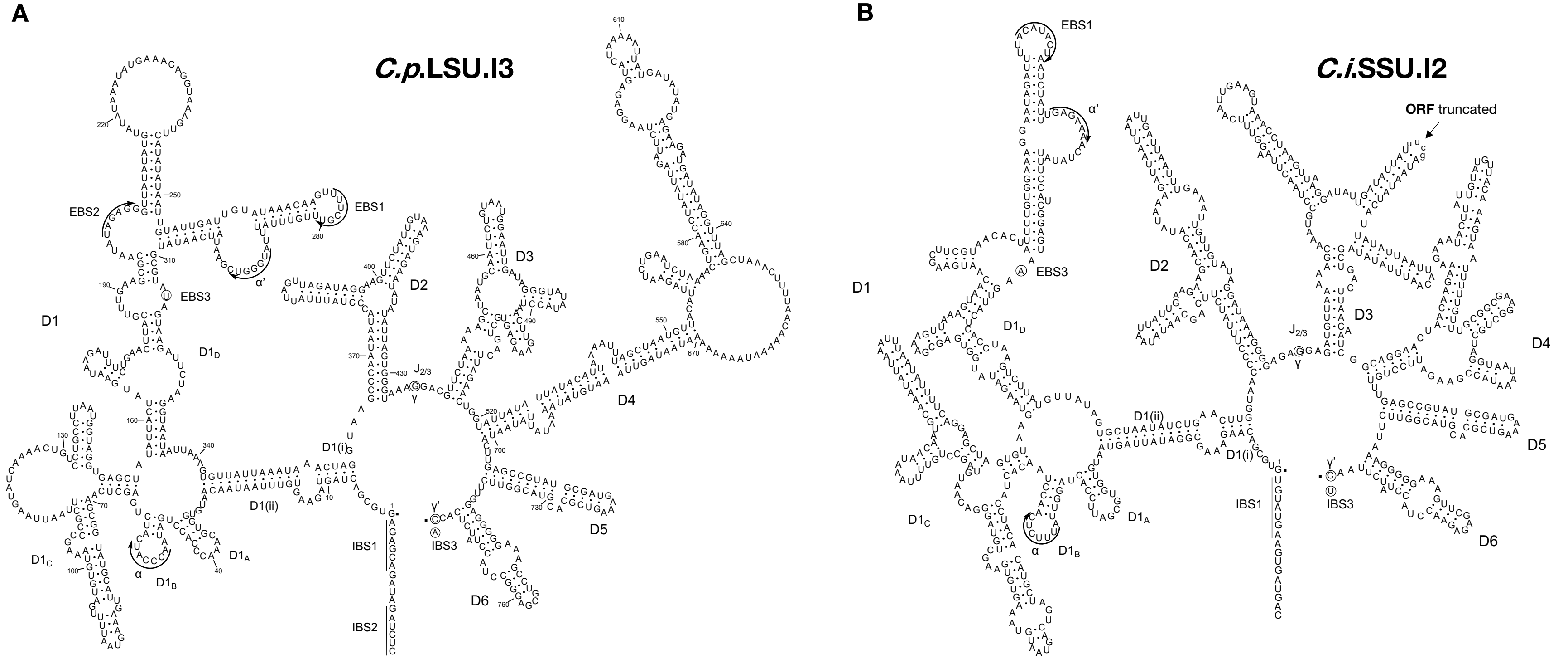

Supplementary Figure 2. Secondary structure prediction of mitochondrial pre-rRNA group II introns found in dimorphic fungi not pursued for *in vitro* studies.

(A) The mitochondrial large subunit rRNA intron found in *Coccidioides posadasii* (*C.p.*LSU.I3).

(B) The second mitochondrial small subunit rRNA intron found in *Coccidioides immitis* (*C.i.*SSU.I2). The intronic ORF is located at domain 3, which has been reduced to an artificial UUCG tetraloop in the secondary structure diagram.

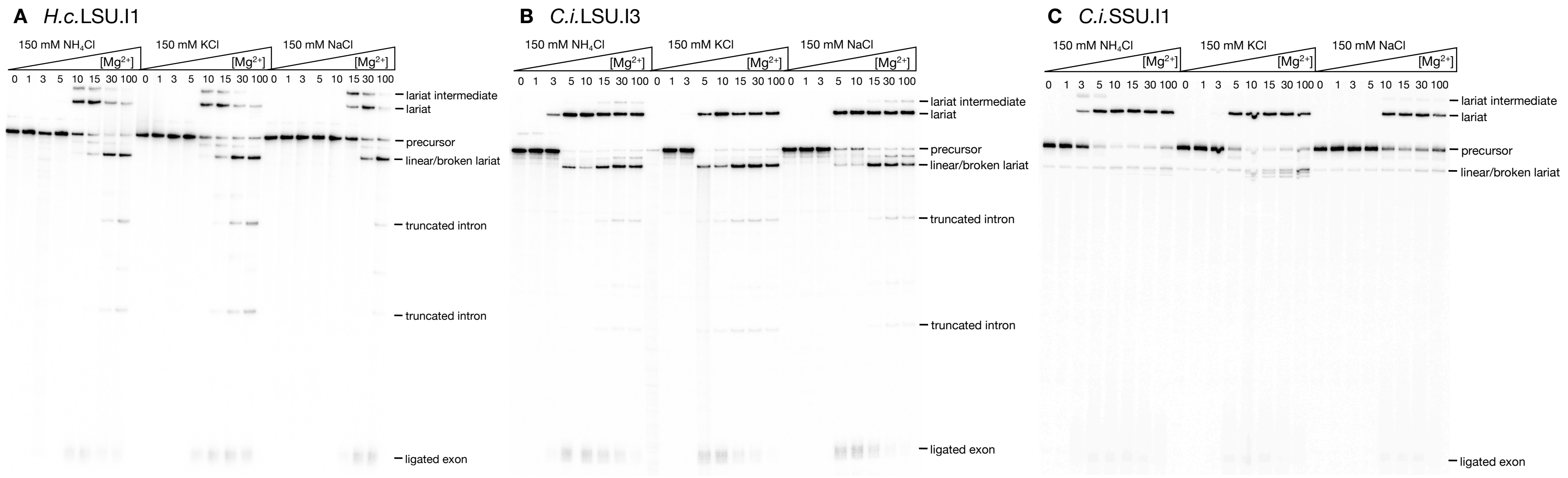

Supplementary Figure 3. Self-splicing outcome under low salt concentration in different monovalent ion conditions with magnesium titration. The reaction is incubated at 37°C for 1 hour in 40 mM HEPES pH 7.5 (pH adjusted with cognate monovalent hydroxide), 150 mM monovalent chloride salt and magnesium whose concentration are indicated on top of the gel lanes.

(A) Splicing outcome of *H.c.*LSU.I1 intron under various conditions. At higher magnesium ion concentrations, broken lariat, which co-migrates with linear intron, and intron truncation products are observed.

(B) Splicing outcome of *C.i.*LSU.I3 intron under various conditions. At higher magnesium ion concentrations, broken lariat, which co-migrates with linear intron, and intron truncation products are observed.

(C) Splicing outcome of *C.i.*SSU.I1 intron under various conditions. In contrast with *H.c.*LSU.I1 intron, neither linear intron/broken lariat nor intron truncation products are observed for the *C.i.*SSU.I1 intron up to 100 mM magnesium ion.

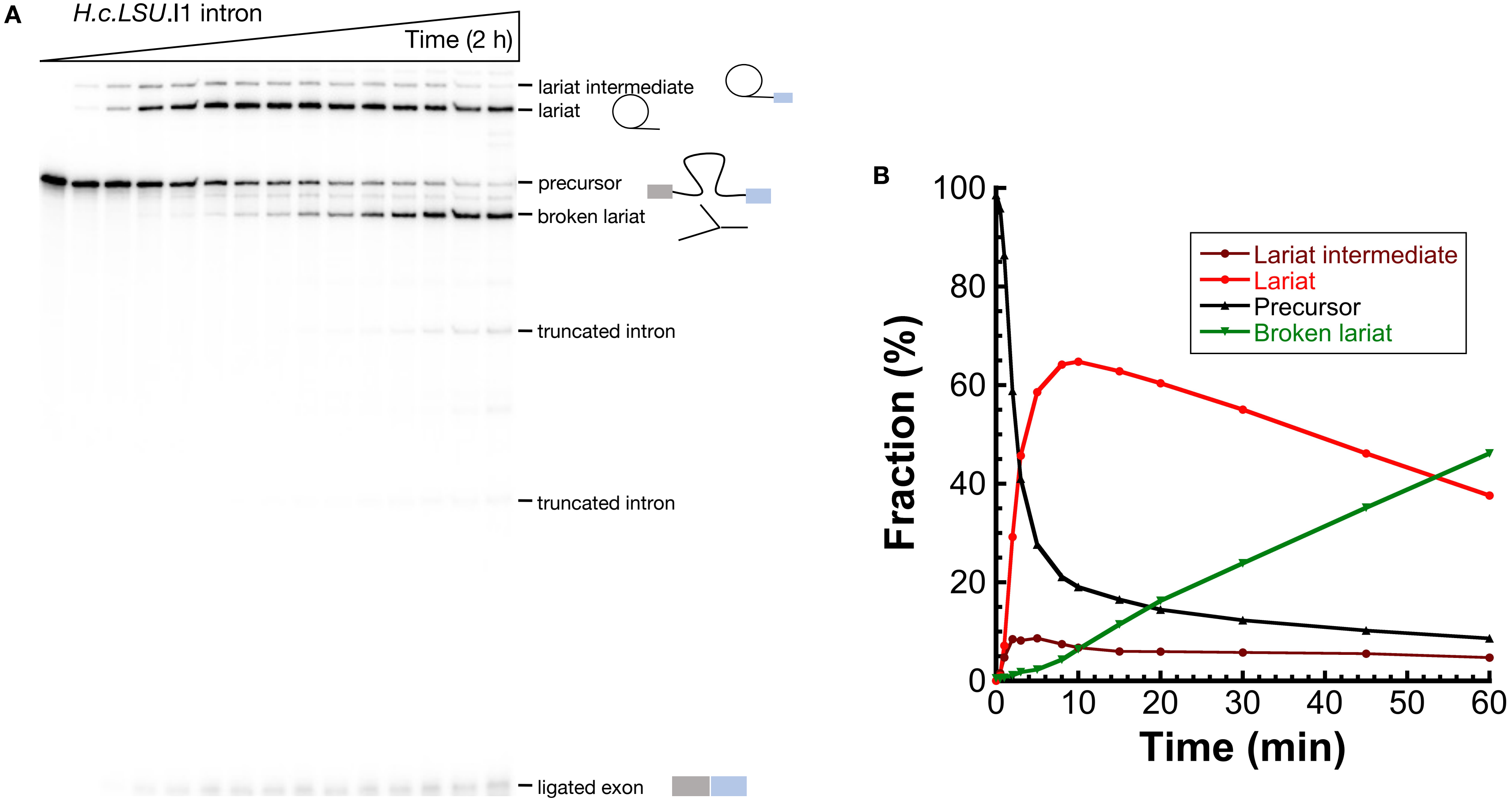

Supplementary Figure 4. The introns have very low intendency to go through hydrolytic pathway.
(A) Splicing time course gel showing the accumulation of intron lariat dominates at early time points with negligible accumulation of linear intron.
(B) Quantitative plot of the splicing time course. The accumulation of apparent linear intron only becomes significant when precursor is almost completely depleted and lariat intron fraction starts to decrease, which suggests that the main source of the apparent linear intron band is broken lariat.

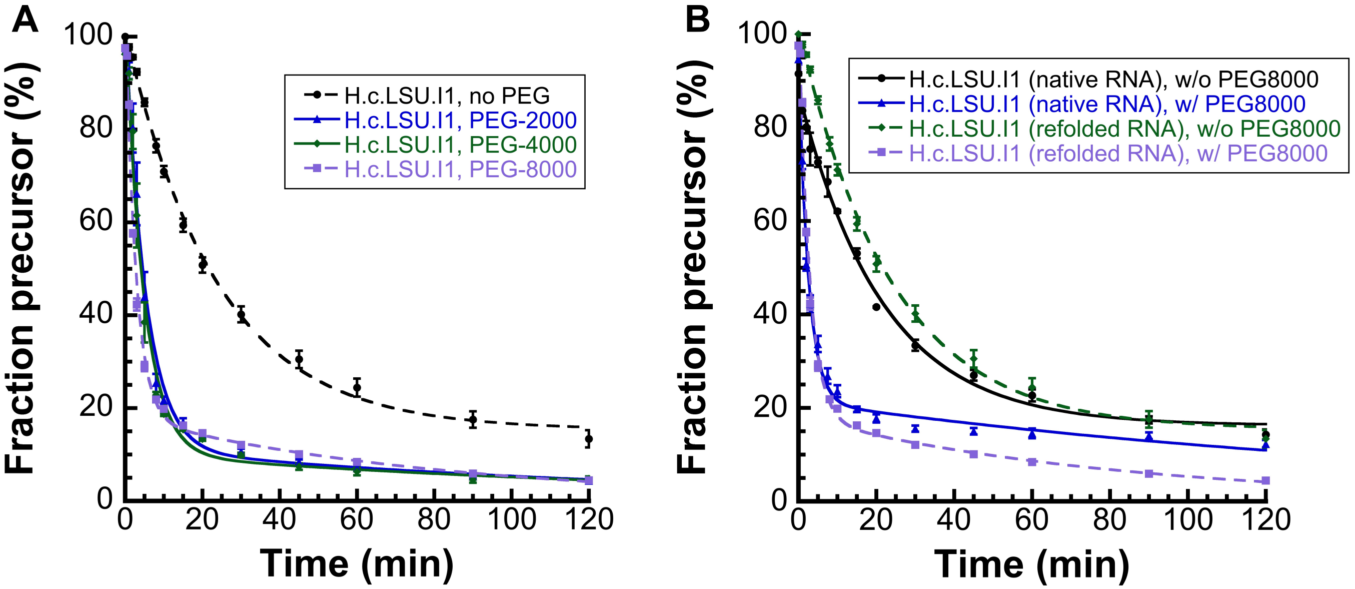

Supplementary Figure 5. Additional characterization of the *H.c.*LSU.I1 intron self-splicing kinetics.

(A) Effect of PEG of different length on the *H.c.*LSU.I1 intron self-splicing kinetics. The reactions are performed under the low-salt condition (40 mM NH_4-HEPES pH 7.5, 150 mM NH_4Cl) at 37°C with no additional PEG (black circle with dashed line), with 10% PEG-2000 (blue triangle with solid line), with 10% PEG-4000 (green diamond with solid line) and with 10% PEG-8000 (magenta square with dashed line). The apparent rate constants are listed in Supplementary Table 5 (Entry 1-4). All three PEG molecules promote self-splicing rate and the self-splicing rate increases with increasing length of the PEG molecules used.

(B) Effect of precursor RNA purification protocols on the *H.c.*LSU.I1 intron self-splicing kinetics. Refolded RNA refers to the precursor RNA purified from the standard denaturing polyacrylamide gel purification scheme as described in the Method section. Native RNA refers to the precursor RNA purified from the non-denaturing size exclusion column directly after *in vitro* transcription*, which is not subject to any denaturation and subsequent refolding step. The reactions are performed under the low-salt condition (40 mM NH_4-HEPES pH 7.5, 150 mM NH_4Cl) at 37°C for both refolded and native RNA without and with 10% PEG-8000. Without PEG-8000, the native RNA (black circle with solid line) gives slightly higher self-splicing rate than the refolded RNA (green diamond with dashed line) (Supplementary Table 5 (Entry 1 and 5)). However, in the presence of 10% PEG-8000, the fast species rate is comparable for both native (blue triangle with solid line) and refolded RNA (magenta square with dashed line) (Supplementary Table 5 (Entry 4 and 6)).

* In brief, cold intron precursor RNA is transcribed from 20 µg of linearized plasmid with in-house prepared T7 polymerase in the transcription buffer described in the method section and 3.6 mM of each NTP. The reaction was incubated at 37`C for 2.5 h and was then subject to DNase I treatment for 15 min to digest the linearized plasmid template. After the incubation, the reaction mixture was buffer exchanged to ME buffer (8 mM MOPS pH 6.5 and 1 mM EDTA pH 8.5) using 50 kDa MWCO Amicon filter to get rid of DNase I and T7 polymerase. The buffer-exchanged sample is further subject to native purification using Superdex 200 Increase 10/300 GL column (GE Healthcare) pre-equilibrated with gel filtration buffer (40 mM NH_4-HEPES pH 7.5 and 150 mM NH_4Cl). Peak fraction was collected and the concentration was measured using Nanodrop 1000. The

final concentration of precursor RNA for the self-splicing reaction was 200 nM in a reaction volume of 40 µL. Self-splicing reaction was then initiated by adding the magnesium chloride stock (10x) to the RNA stock. The reaction was incubated at 37°C and a small volume (2 µL) was removed from the reaction mixture and quenched by mixing with 2x formamide loading dye. The timepoint samples were then loaded onto the 5% denaturing polyacrylamide gel to resolve individual bands. The gel was stained by GelRed (Biotium) and imaged with Typhoon RGB Biomolecular imager (Cytiva) using the Cy3 channel. Individual bands were quantified using ImageQuant 8.2 software (GE Healthcare). The band intensity was corrected for the length of corresponding species and internally calibrated by calculating the fraction of each species in the reaction. The precursor depletion time course was then analyzed using the equations described in the method section.

Supplementary Table 1. Survey of group II introns in the mitochondrial genomes of human pathogenic fungi by conserved D5 domain.

(*See individual spread sheet*)

Supplementary Table 2. Sequences and folding constraints of group II introns discovered in this study.

Intron	Sequence	mfold folding constraints
*H.c.*LSU.I1	GUGCGACUAGUAAAGUGUUUAAUAAUAAUGUGGUGAAAACCCACCAGAUAACCCAU<br>UAUCUGAGCUCAUACGGUGUGCAUUAAAAGUAAUUUUUUUGUAUAAAGCCCGUUUA<br>AUCAGGUAAAUAAUCCUUCCAUCCUAAUUAUAUCUAUAGAUAUAAAAGGAUGGUGA<br>GCUAUAUUACUAUGGAUAAGAUUUUUUUGAAUUCACGUUUGAAGCGUAUUAGAGUA<br>AGUUUUAUUUAAAAGGAAAAAAAAAAAAUUAAAUAAAAUUAAAUUAAUUGUAUAAAUAA<br>GUUUUCGUUUAUUUAUUAAUGGGUCUUAUAUUAAUGUACGUAUAGUGAAAUCCUAA<br>UGUAGUAAUUAAGGUGUUAUUAAAUAAACUAGGUAAGCCCAAUAAUGUCUUCAUAA<br>UAUGAAGAAGUUCAAGUGUAAAUUUGAAUAUACAUUAGUGGGUAAAGGAUAUUUUA<br>AAAAGCGAAUGUCUCAUAUUAAUAGUGAGAAUAGGUCUAUGACUAAUUCGAAAGAA<br>UGCUGACUUAAAAUUAAUAUUAAUAUUAAUAUAUAUAUAUUAAUAUUUGAGCCGUAU<br>GCGAUGAAAAUCGCACGUACGGUUCUUAGAGGGGGAAAGCUCGAGAGGGCCUACC<br>UAUCUCCAC	P 1 0 6, P 28 0 4, P 51 0 6,<br>P 86 0 4, P 102 0 4, P 197 0 3,<br>P 217 0 7, P 285 0 7, P 301 0 6,<br>P 348 0 4, F 7 370 5, F 16 364 12,<br>F 278 299 7, F 266 317 6,<br>F 273 311 3, P 371 0 4, P 438 0 7,<br>P 521 0 7, P 554 0 1,<br>P 578 0 2, P 589 0 3
*B.d.*LSU.I2	GUGCGACUAGUAAAGUGCUUAAUAACAAUGUGGUGAAAGCCCACCAGAUAACCCAU<br>UAUCUGAGCUCAGACGGUAUGCAUUAGAAGUAAUUCUUUUGUGUAAAGCCCGUUUA<br>AUUUGGUAAACAGACCAACCAACCUAUAUAUAAGGAUGGUGAGCUAUAUUACUAUG<br>GAUAAAAUUUUUAUGAAUUCACGUUCGAAGCGUAUUAGAGUGAGUUUUAUUUAGAA<br>GGAAAAAAGUAAAUAAAAUUCUAAUUAAUUGUAUAAACAAUUUUUCGUUUGUUUAUU<br>AAUGGGUCUUAUAUUAAUGUACGUAUAGUGAAAUCCUAAGGUAGUAAAUAAGGUGU<br>UAUUAAGUAAACUAGGUAAGCCCAAUAAUAUCUUCAUAUGAUAGUAUGAAGAAGUU<br>CAAGUGUAAAUUUGAAUAUAUAUUAGUGGGUAAAGGAUAUUUUAAAAAGCGAAUGU<br>CUCAUAUUAAUAGUGAGAAUAGGUUUAUGACUAAUUCGAAAGAAUGCUGACUUAAA<br>AUUAAUAUUAAUAUuucg[*]AUAUUAAUAUUUGAGCCGUAUGCGAUGAAAGUCGCACGU<br>ACGGUUCUUAGAGGGGGAAAGUCCAAGAGGGCCUACCUAUCUCAAC	P 1 0 6, P 52 0 4, P 183 0 3,<br>P 204 0 6, P 267 0 6, P 285 0 4,<br>P 353 0 4, P 425 0 7, P 559 0 2,<br>P 570 0 3, F 469 482 3,<br>F 484 493 3, F 536 569 4
*C.i.*LSU.I3	GUGCGACUAGAUAAGUACUUAAUAGCAAUGUGGUGAAAGUCCACCUGAUAACCCAU<br>AGUCUGAGCUCAGACGGUAUGCAUUAGAAGUAAUUCUUUUGUGUAAAGCCCGUUU<br>AAUCUGGUCUAAAGGACCUUCCACCCUAAAUAUAUAGGGGGAGAGCUAUAAUACUA<br>AGGAUAAAAUUUUUUUGAAUUCAUGUUUAAAGCGUAUUAGAGGAAAUUUAUUUCUA<br>UGAAAAAGAAGAAAUAAAUUUUUAAUUAACUGUAUAAACAAGUUUUCGUUUGUUUAU<br>UAGUGGGUCUUAUGUUAAUGUACGUAUAGUGGAAUCCUAAGGUAUUAAUUAAGGU<br>GUUAUUAAGUAAACUAGGUAAGCCCAAUAGUGUCUUCAUAUUAUAAUAUGAAGAAG<br>UUCAAUUGUGAAAUUGAAUAUGCAUUAGUGGGUAAAGGAUAUUUUAAAAAGCGAAU<br>GUCUCAUAUUAAUAGUGAGAAUAGGUCUAUGACUAAUUCGAAAGAAUGCUGACUUA<br>AAAUUGGUAUUAAUAUAUGCGUuucg[†]AUGUAUAUAUUAAUAUUUGAGCCGUAUGCG<br>AUGAAAGUCGCACGUACGGUUCUUAGAGGGGGAAAUCUUAGUAAUAAGACGACCUA<br>UCUCGAC	P 51 0 6, P 184 0 3, P 205 0 6,<br>P 267 0 6, P 283 0 6, F 47 60 3,<br>P 353 0 4, P 425 0 7, P 571 0 2,<br>F 432 507 7
*C.i.*SSU.I1	GUGCGACUUGUUAAGUUUUAACAAAAAUUGUAUAACGUUUAUUAAUGAUUAUACAU<br>UGUAUUUCAUCUUACAAUAGCCUAAUUAGAUAUGCAUUUAGGGUAACUUUUUUGUA	P 62 0 6, F 164 171 2, P 172 0 3,<br>P 215 0 6, P 232 0 6, P 297 0 4,

	UAAAGCUCUAAUUAUAAGUGUAAAUACACUUUUAGGCUUCUUCUAUGGUUAGAGAA<br>AUCGAACCAAUGUAAUUAAAACUUUGAUGUAUUAGGCAUUUAACGUGUCCUUGGUU<br>AAAUGAAGAUGAACAUAAGUAUACAAAGUAAAAUUGGAACCUAAGGAAGAAUUGUUU<br>UUGUUAAGAAACAAGGUAAUACCUAUAACUGGCUAAUAUAAAUUUGCAAGGUUUAU<br>UGUAAAAUAAACUAUAGGUUAGAGGUAAAAGGAUAAUGUAAAAAGCGAAUGCAAUU<br>CUGUAAUGGAAUUGAUAGGGUAUAUACCUAACUUGAAAGAGUGCUGACUUACAUAU<br>AGAUGUUAUUUACGUuucg[†]ACGUAAAUAAUGUUUGAGCCGUAUGCUAUGAAAGUAG<br>CACGUACGGUUCUAAGAGGGGGAAAGUCCGAGAGGACCUACCUAUCUCAAC	P 365 0 8
*C.i.*SSU.I2	GUGCGACAAGAAAGCGGAUAUUAGUAAUGUGGUGCGAUUCCACUAGGUUAUUUCU<br>CAACCUAACACUACCUACACAUGCUAGUCAGUAAUGUAAAGUGUGAAGCUGUAGGG<br>ACAAUGUAGCCUGUUUUAAAUAACAAUGCAAAUAUUAAAUUUAAUAUUUUCAGGAGC<br>UAGUGUAAGUAAGAUAUGGUGAGCGAAAGUUAAAGUAACAAUGAAGCUUCGUAACA<br>CUAUUUUGUGGAAAGGAAUAGAUUUUACAUACUAAUCUAUUUGAGAAAACUAUAUU<br>UCCAUCGGAGUAAAGUUACUCACCUAAGUCUUAUGUUAUAGUGCUAAUAUCUGAAC<br>UUGGUAACCCCUUUAUCUUCAGCAAUAUAAUUAUUGAAGAAGCAACAUAUAAAGAU<br>UAAUUAAUUGAUUAAUUUGAAAUUGUUGUAUAGGAUAAAGGGGAGAGGAGAUGUUA<br>AAAAGCAAAUGCCUAACUUAAGGUUUCAAUUGAAGUAAACCUAAGUUAAGGAUAUU<br>GAUAUUAUuucg[†]AUAAUAUCAUUAUAUUAAUUACAAUUUAUAUAGCUGACUUAACAA<br>UCGGCAGGAACUAACAAGAAAGAAAUACUUAUUGUAUGUUACAAAGUAAUUUUUGU<br>UUGCGGCGAAGGCUGCUAGGUAAUAAAUACCGAAGAUUCCUGUUUGAGCCGUAUG<br>CGAUGAAAGUCGCACGUACGGUUCUUAAAGGGGGAAAGUUCGAGAGAACCUACCU<br>AUCUUAAC	P 51 0 6, P 251 0 6, P 266 0 6,<br>P 292 0 3, P 340 0 4, P 436 0 6,<br>F 527 548 5, P 564 0 1, P 661 0 2,<br>P 686 0 2, P 697 0 3
*C.p.*LSU.I3	GUGCGACUAGUAGAAGUGUUUAAUAACAAUGUGGUGCAAACCCACCUGAUAACCCA<br>UCAUCUGAGCUCAAGCGGUAUGCAUUGAAAGUAAUUUUGAUGUGUAAAGCCCGCU<br>UAAUUAAGUAUCAAAACUGUCCUGCCUUAAUGGUAGGUGAGCUAUAUUACUAUGAA<br>UAAGAUUUCUGAACUUACGUUUGAAGCGCAAUAUAGAGGGUUAUAUAUGUAUAUAA<br>AUAUGAAACAGGUAAAGUUCAUAUAUAAUUGUAUUGAUUGUAUAAACAAGUUUUCG<br>UUUGUUUAUUUAUGGGUCGAAUAUCAAUAUGCGUAUAGUAAGAUUCUAAGGUAAUA<br>AUUAAAGUGUUAUUAAAUAAACUAGGUAAGCCCAAUAAUACCCUAUUUAUUAGUUAG<br>AUAGGAAGUUCUAUUGUAAAGUAGAAUAUAUAUUAGUGGGUAAAGGACGUUCUAAA<br>AAGCUAAUGCAAUUCUGUAAUGGAAUUGAUAGGGUAUAUACCUAACUUGAAAGAGU<br>GCUGACUUAGAACUGGUAUUAUAUUAUACAUUAAAAUUUAGCUAAUGUUACAUUAG<br>AAUCUGAAUCUAAAACUGAACCUAUAUUAGAUUCUAAGGAGAGUACUAAAAAUUAUG<br>AUAUAUGAGAAGAUGAUAUAGGUUUAGCUAAACUUUUAACAAAAAUAAAAAAAAUAA<br>UAGUUAAAUGUAUAAAAUAUUAUAAUACUUGAGCCGUAUGCGAUGAAAGUCGCACG<br>UACGGUUCUUGGAGGGGGAAAGCCUGCGAGGGCCUACCUAUCUCACC	P 1 0 6, F 48 61 3, P 52 0 6,<br>P 178 0 3, P 201 0 6, P 275 0 6,<br>P 291 0 6, P 314 0 3, P 361 0 4,<br>P 434 0 7, P 518 0 3, P 702 0 3,<br>P 728 0 2, P 739 0 3, P 775 0 3

[*] The basal stem of D4 is intact and the extra sequence of D4 is trimmed to the UUCG tetraloop for the design of self-splicing construct.
[†] The original sequence of the intronic open reading frame (ORF) is reduced to the UUCG tetraloop for the design of self-splicing construct.

Supplementary Table 3. Intron presence survey across fungal strains with available genome assembly.

Organism	Search intron	Strain	Presence	Mitochondrial genome contig assembly GenBank accession
B. dermatitidis	*B.d.*LSU.I2	ATCC 18187	√	AJJV01017395
		ATCC 18188	√	ADMK01007373
		ATCC 26199	√	AEII01004165
		ER-3	√	ACBT01000592
B. gilchristii	*B.d.*LSU.I2	SLH14081	√	ACBU01001792
H. capsulatum	*H.c.*LSU.I1	H88	√	ABRJ01000463
		H143	√	ABRK01004267
		G186A	×	JABBCE010000001
		NAm1	√	AAJI01002868
		G217B	Mitochondrial genome not available*	
C. immitis	*C.i.*LSU.I3	RS	√	AAEC03000011
		WA_211	√	RHJW02000007
		H538.4	√	AASO01004342
		RMSCC 3703	√	ABBC01005031
		RMSCC 2394	√	AATX01000518
	*C.i.*SSU.I1	RS	√	AAEC03000011
		WA_211	√	RHJW02000007
		H538.4	×	AASO01004342
		RMSCC 3703	√	ABBC01005031
		RMSCC 2394	√	AATX01000518
	*C.i.*SSU.I2	RS	√	AAEC03000011
		WA_211	√	RHJW02000007
		H538.4	×	AASO01004342
		RMSCC 3703	√	ABBC01005031
		RMSCC 2394	√	AATX01000518
C. posadasii	*C.p.*LSU.I3	CPA 0001	√	ABFO01004995
		CPA 0020	√	ABIV01004481
		CPA 0066	√	ABIW01004265
		RMSCC 1037	√	ABIS01004316
		RMSCC 1038	Mitochondrial genome not fully assembled†	
		RMSCC 2133	√	ABFM01001051
		RMSCC 3488	√	ABBB01000285
		RMSCC 3700	Mitochondrial genome not fully assembled†	
		C735 deltaSOWgp	√	ACFW01000039
		Tuc2	√	MW722165 (1)
		Silveira	Mitochondrial genome not available*	

*C.p.*SSU.I1	CPA 0001	√	ABFO01004995
	CPA 0020	√	ABIV01004518
	CPA 0066	√	ABIW01004269
	RMSCC 1037	√	ABIS01004316
	RMSCC 1038	√	ABIT01004892
	RMSCC 2133	√	ABFM01001051
	RMSCC 3488	√	ABBB01000285
	RMSCC 3700	Mitochondrial genome not fully assembled†	
	C735 deltaSOWgp	√	ACFW01000039
	Tuc2	√	MW722165 (1)
	Silveira	Mitochondrial genome not available*	

The intron sequence from reference genome is subject to BLASTN search algorithm against NCBI whole-genome shotgun contigs database. The criteria for the presence of corresponding intron are: 1) whether a specific strain has a hit that has >80% query coverage and 2) >95% identity value.

* To ensure the coverage of mitochondrial genome, part of *cox1* gene exon sequence is subject the same BLASTN search and criteria. If no significant hit meeting the criteria, the mitochondrial genome is perceived as not available for a specific strain and therefore unable to infer the presence or absence of the corresponding of the intron.

† To further ensure the coverage of *LSU* gene, the exon sequence flanking 5' end of the intron is subject to the same BLASTN search and criteria. If no significant hit meeting the criteria, the mitochondrial genome is perceived as not fully covered and therefore unable to infer the presence or absence of the corresponding of the intron.

Supplementary Table 4. Estimation of the minimal [Mg^{2+}] requirement for the representative fungal group II introns to self-splice.

Entry	Intron	Additional salt	PEG8000	[Mg^{2+}]$_{minimal}$ (mM)
1	*H.c.*LSU.I1	150 mM NH_4Cl	none	10
2	*H.c.*LSU.I1	150 mM NH_4Cl	10%	3
3	*H.c.*LSU.I1	150 mM KCl	none	10
4	*H.c.*LSU.I1	150 mM NaCl	none	15
5	*B.d.*LSU.I2	150 mM NH_4Cl	none	10
6	*B.d.*LSU.I2	150 mM NH_4Cl	10%	3
7	*C.i.*LSU.I3	150 mM NH_4Cl	none	5
8	*C.i.*LSU.I3	150 mM NH_4Cl	10%	3
9	*C.i.*LSU.I3	150 mM KCl	none	5
10	*C.i.*LSU.I3	150 mM NaCl	none	10
11	*C.i.*LSU.I3	none	10%	0.5
12	*C.i.*SSU.I1	150 mM NH_4Cl	none	5
13	*C.i.*SSU.I1	150 mM NH_4Cl	10%	3
14	*C.i.*SSU.I1	150 mM KCl	none	5
15	*C.i.*SSU.I1	150 mM NaCl	none	10
16	*C.i.*SSU.I1	none	10%	0.3

All reactions are in 40 mM NH_4-HEPES pH 7.5 buffer and incubated at 37°C; presence of any additional salt and PEG-8000 are indicated in the table. To get an estimation of the minimal [Mg^{2+}] requirement, we titrated the following [Mg^{2+}]: 0.1, 0.3, 0.5, 1, 3, 5, 10, 15 and 30 mM. The minimal magnesium ion concentration at which the precursor has converted greater than 50% after incubation at 37℃ for 1 hour is reported.

Supplementary Table 5. Self-splicing kinetics of representative fungal group II introns under various conditions.

Entry	Intron	Ionic condition		Crowding agent	Precursor conversion kinetics*				
		$[NH_4Cl]$ (mM)	$[MgCl_2]$ (mM)		Kinetic Phase(s)	f_{fast} (%)†	k_{fast} (min^{-1})‡	f_{slow} (%)	k_{slow} (min^{-1})
1	*H.c.*LSU.I1	150	10	none	1	86.1 ± 1.1	0.039 ± 0.001	/	/
2	*H.c.*LSU.I1	150	10	10% PEG2000	2	92.6 ± 5.1	0.19 ± 0.02	11.3 ± 4.8	0.007 ± 0.008
3	*H.c.*LSU.I1	150	10	10% PEG4000	2	93.6 ± 5.5	0.21 ± 0.03	10.2 ± 5.0	0.007 ± 0.009
4	*H.c.*LSU.I1	150	10	10% PEG8000	2	86.6 ± 4.2	0.36 ± 0.04	18.0 ± 3.5	0.012 ± 0.005
5	*H.c.*LSU.I1§	150	10	none	1	71.6 ± 1.7	0.046 ± 0.003	/	/
6	*H.c.*LSU.I1§	150	10	10% PEG8000	2	73.2 ± 2.5	0.39 ± 0.03	21.4 ± 1.6	0.006 ± 0.002
7	*C.i.*LSU.I3	150	5	none	1	88.7 ± 1.5	0.75 ± 0.03	/	/
8	*C.i.*LSU.I3	150	5	10% PEG8000	1	100.8 ± 5.6	2.00 ± 0.22	/	/
9	*C.i.*LSU.I3	0	0.5	10% PEG8000	1	85.9 ± 2.1	0.28 ± 0.02	/	/
10	*C.i.*LSU.I3	0	1	10% PEG8000	1	100.2 ± 3.2	0.28 ± 0.03	/	/
11	*C.i.*LSU.I3	0	3	10% PEG8000	1	94.8 ± 2.5	0.40 ± 0.03	/	/
12	*C.i.*LSU.I3	0	5	10% PEG8000	1	99.7 ± 2.8	0.41 ± 0.03	/	/
13	*C.i.*SSU.I1	150	5	none	1	97.4 ± 1.7	0.43 ± 0.02	/	/
14	*C.i.*SSU.I1	150	5	10% PEG8000	2	73.6 ± 2.2	2.94 ± 0.18	26.0 ± 1.9	0.18 ± 0.03
15	*C.i.*SSU.I1	0	0.5	10% PEG8000	1	88.2 ± 1.4	0.33 ± 0.02	/	/
16	*C.i.*SSU.I1	0	1	10% PEG8000	1	96.1 ± 1.9	0.51 ± 0.03	/	/
17	*C.i.*SSU.I1	0	3	10% PEG8000	2	76.8 ± 3.5	0.48 ± 0.05	26.0 ± 3.5	0.016 ± 0.008
18	*C.i.*SSU.I1	0	5	10% PEG8000	2	63.4 ± 2.8	0.41 ± 0.03	39.6 ± 2.9	0.020 ± 0.004

All reactions are in 40 mM NH_4-HEPES pH 7.5 buffer and incubated at 37°C; any additional salt, magnesium ion concentration and the presence of PEG-8000 are indicated in the table.

* The kinetics are reported in terms of precursor conversion, which yield apparent rate constants.

† For monophasic kinetics, the data reported in this column are the fraction of actively converting precursor

‡ For monophasic kinetics, the data reported in this column are the rate of actively converting precursor

§ Natively-purified intron precursor RNA (not subject to any denaturation or refolding step).

Reference

1. de Melo Teixeira, M., Lang, B.F., Matute, D.R., Stajich, J.E. and Barker, B. (2021) The mitochondrial genomes of the human pathogens Coccidioides immitis and C. posadasii. *G3 (Bethesda)*.
